# A pilot study on a combined non-invasive screening test for metabolic dysfunction-associated steatotic liver disease and type 2 diabetes

**DOI:** 10.3389/fendo.2026.1780005

**Published:** 2026-02-12

**Authors:** Katrin Saenger, Christian Torres Reyes, Oscar Cahyadi, Alanna Ebigbo, Wolfgang Ekkehard Schmidt, Daniel Robert Quast

**Affiliations:** Department of Internal Medicine I, St. Josef-Hospital, Ruhr-University Bochum, Bochum, Germany

**Keywords:** diabetes, MASLD, metabolic disease, methionine kinetics, non-invasive test, oral glucose

## Abstract

**Background:**

Type 2 diabetes (T2D) and metabolic dysfunction-associated steatotic liver disease (MASLD) are increasing globally, with mitochondrial dysfunction being a core component in their development. While a 75g oral glucose tolerance test (OGTT) can be used to diagnose T2D, hepatic metabolic and mitochondrial dysfunction can be assessed using a ^13^C-methionine breath test (BT). We aimed to evaluate combining these tests for an efficient, non-invasive screening tool.

**Methods:**

On three study days, 26 subjects (11 [43.3%] female, 61± 16 years) subjects underwent either an OGTT, a ^13^C-methionine BT or both tests combined. Diagnostic outcomes of the individual and combined tests were compared using cumulative ^13^C percentage dose recovered (cPDR), plasma glucose concentrations and Homeostatic Model Assessment (HOMA)-indexes for insulin resistance and beta-cell function.

**Results:**

In the combined test, cPDR_90min_ was significantly lower (cPDR_90min_ 2.3± 0.2% vs. 5.7± 0.5%; p< 0.0001), accompanied by a rightward shift of the ^13^C-increase towards later time points. When breath collection of the combined test was extended, cPDR_145min_ (5.7± 0.4%) was practically identical to cPDR_90min_ of the single test (p= 0.99). OGTT results, plasma glucose, and HOMA-indexes did not differ significantly between tests.

**Conclusions:**

Combining a ^13^C-methionine BT with an OGTT significantly impacts ^13^C-methionine kinetics, but not OGTT results. A potential mechanism includes a glucose-induced delay of gastric emptying. Combined testing may be feasible when time-adjusted measurements are used, potentially allowing simultaneous screening for both T2D and MASLD-associated mitochondrial dysfunction in clinical practice.

## Highlights

- Hepatic mitochondrial dysfunction is central to diabetes and MASLD pathogenesis.
- Oral glucose significantly delays ^13^C-methionine breath test kinetics.
- Extending the sampling period in combined testing yields comparable results.
- Diagnostic outcomes of the oral glucose tolerance test and HOMA remain unchanged.
- A combined approach enables efficient integrated assessment for metabolic disease.

## 1 Introduction

Metabolic diseases are an increasing global socioeconomic burden characterized by a high incidence and mortality (1). Obesity, type 2 diabetes (T2D), metabolic dysfunction-associated steatotic liver disease (MASLD) and cardiovascular disease share several factors in their pathogenesis and represent the majority of cases with metabolic diseases worldwide (1, 2). Both conditions independently increase carotid intima-media thickness, a marker of subclinical atherosclerosis, through systemic low-grade inflammation, insulin resistance, and pro-atherogenic cytokines like IL-6 and TNF-α (3, 4) and significantly increase cardiovascular risk (5). Patients presenting with either T2D or MASLD have a more than twofold increased risk of developing the other, suggesting that in clinical practice, patients with one condition should be screened for the other (6). However, the clinical assessment of metabolic disease and its associated risks can be challenging, especially in borderline cases.

Beyond their independent associations with obesity, both T2D and MASLD are characterized by progressive hepatic mitochondrial dysfunction (7). Recent evidence demonstrates that impaired mitochondrial β-oxidation and oxidative stress represent critical elements in the pathophysiology of both conditions, suggesting that simultaneous assessment of glucose homeostasis and hepatic mitochondrial function could provide integrated mechanistic insights into metabolic disease pathogenesis (8, 9). Hepatic mitochondrial function can be assessed non-invasively *in vivo* using a ^13^C-methionine breath test (10). This test can stratify hepatic mitochondrial function and is potentially able to differentiate between MASLD, metabolic dysfunction-associated steatohepatitis (MASH) and MASH cirrhosis (11, 12). In contrast, screening for T2D is often performed using point-of-care plasma glucose or glycated hemoglobin (HbA_1c_) analysis. However, the American Diabetes Association also recommends a 75g oral glucose tolerance test (OGTT) as first or second line (e.g., in subjects with a HbA_1c_ of 5.7 – 6.4%) diagnostic in subjects with suspected diabetes (13). Given the strong correlation of diabetes with other metabolic diseases and cardiovascular disease, screening for other conditions like MASLD in these subjects could improve early diagnosis and, potentially, patient outcomes. Compared to transient elastography or liver biopsy, both the ^13^C-methionine breath test and the OGTT are simple and inexpensive tools to screen for the presence of metabolic diseases and are characterized by their capability to do detect even early stages of the disease or dysfunction, respectively. Nonetheless, this cost-effectiveness applies to well-equipped clinical settings but is limited by ^13^C-isotope availability globally. Moreover, subjects must be fasted for both tests which can hinder their implementation, especially in outpatient settings. Performing these tests simultaneously has not been examined yet. The primary objective of the present study is therefore to evaluate whether combining the two tests alters methionine kinetics and whether time-adjusted measurement might recover comparability to single-test protocols to evaluate if this approach could potentially offer an efficient non-invasive screening tool for metabolic assessment.

## 2 Materials and methods

The present study was a monocentric pilot study performed from February to October 2023 at the Department of Internal Medicine of the St. Josef Hospital, University Hospital of the Ruhr University Bochum, Germany. The study was conducted according to the principles of good clinical practice and approved by the local ethics committee before commencement (registration no. 22-7723). Written informed consent was obtained from all subjects before any study procedures were conducted.

### 2.1 Study population

In- and outpatients treated at St. Josef-Hospital Bochum and its adjunct outpatient facilities were eligible if a physician requested either a ^13^C-methionine breath test or an oral glucose tolerance test (OGTT). The decision to perform either test was made at the physician’s discretion and independent from the study. All potentially eligible subjects were screened for inclusion after scheduling either test, with consecutive recruitment to reduce selection bias. Major inclusion criteria for the study was a body mass index (BMI) ≥ 25 kg/m^2^. Subjects who exhibited conditions that could potentially affect the absorption of ^13^C-methionine or glucose from the gastrointestinal tract or affect the exhalation (14) were excluded from the study. Full inclusion and exclusion criteria are reported in **Supplementary Table S1**.

### 2.2 Screening visit

Participants underwent a physical examination including evaluation of laboratory parameters and determination of the FIB-4 score (15). A thorough medical history was obtained with a focus on cardiovascular, metabolic, and liver conditions.

### 2.3 Interventions

Following the initial screening, participants were invited to three study visits. Each visit, subjects underwent either a ^13^C-methionine breath test, an OGTT, or a combined test involving both a ^13^C-methionine breath test and an OGTT. The test sequence always started with the test requested by the physician (either ^13^C-methionine breath test or OGTT) and was determined by the study personnel. The order of the remaining tests was at random. Subjects were invited to the study site fasted and refraining from nicotine or alcohol for at least eight hours. For the ^13^C-methionine breath test and the combined test, subjects were asked to avoid ^13^C-rich food for 2 days (e.g. corn, pineapple, broccoli, sugarcane). For the OGTT and the combined test, participants were instructed to consume a diet containing at least 150 g carbohydrates per day for the three days leading up to the test.

### 2.4 ^13^C-methionine breath test

The ^13^C-methionine breath test is a non-invasive method to assess the mitochondrial function of hepatocytes and may indicate metabolic dysfunction-associated steatohepatitis (MASH) (14, 16). In this test, 2 mg methionine per kg body weight are dissolved in 100 ml of water. Gas-tight sampling breath test bags are prepared, and breath samples are collected every 10 minutes over a 90-minute period (total of 10 bags, t_-5_ – t_90_). The analysis is performed on-site by isotope-selective nondispersive infrared spectroscopy (IRIS^®^ system). A baseline breath sample is taken before drinking the ^13^C-methionine solution. The primary results are expressed as the delta of the ^13^C/^12^C-concentration over the baseline (DOB). To quantify the metabolized substrate, the results are expressed as ^13^C percentage dose recovered (PDR) for each time interval and the cumulative ^13^C percentage dose recovered (cPDR_90min_) after 90 minutes. While the ^13^C-methionine breath test primarily assesses hepatic mitochondrial function, some studies suggest that it has potential for the non-invasive diagnosis of MASH (14). In this context, a cPDR_90min_ of more than 4.2% indicates the absence of MASH. Values below 4.2% suggest the presence of MASH, and those below 3.65% are suggestive for liver fibrosis of higher degree (F2-F3) (14).

### 2.5 Oral glucose tolerance test

The OGTT is a test used to detect impaired glucose metabolism and even early stages of diabetes mellitus. The test was performed in adherence to the current recommendations of the American Diabetes Association (13). A peripheral venous catheter was placed, and subjects were placed in a sitting or reclined position, with the subject refraining from physical activity.

At baseline, two blood samples were drawn before consuming the glucose solution (t_-5_ and t_0_). Then, a solution containing 75g of glucose dissolved in 300 ml of water was administered orally (t_0_). Further blood samples were collected at 60 minutes (t_60_) and 120 minutes (t_120_) after oral glucose administration. These blood samples were centrifuged and analyzed for plasma glucose, insulin, and C-peptide. Homeostasis Model Assessment (HOMA) for insulin resistance (HOMA-IR) and β-cell function (HOMA-B) were determined as previously described (17–19).

### 2.6 Combined ^13^C-methionine breath test and oral glucose tolerance test

The combined test was performed similar to the procedures outlined above. However, breath samples were collected every 10 minutes over an extended duration of 180 minutes (instead of 90 minutes) to account for potential delayed increase in ^13^C concentration (e.g., due to glucose-mediated deceleration of gastric emptying) (20). Aside from this, the test protocol remained unchanged.

### 2.7 Safety and adverse events

Patients received detailed informed consent regarding potential adverse effects including dizziness, nausea, sleepiness, polyuria, or blood pressure changes. Subjects were monitored during all test procedures. Adverse events were systematically documented.

### 2.8 Endpoints

The primary endpoint was to determine if there is a difference of ≥ 10% between the ^13^C-methionine breath test and the combination of an 75g oral glucose tolerance test and a ^13^C-methionine breath test in the cumulative ^13^C percentage dose recovered (cPDR) at t = 90 min (cPDR_90min_). We assumed from previous studies that the caloric load of the OGGT would decelerate gastric emptying velocity (21) and speculated that this would result in a delayed ^13^C exhalation in the combined test. Consequently, the secondary endpoint was to determine the time (t_x_) at which the cPDR of the combined test was equal to the cPDR_90min_ of the ^13^C-methionine breath test without OGTT.

### 2.9 Statistics

#### 2.9.1 Power calculation

Pre-study sample size was estimated using G*Power 3.1.9.7 (22). A study of our group showed a cPDR_90min_ of 6.15 ± 1.2% for healthy controls (14). For the primary endpoint, the ^13^C-methionine breath test and the combined test were assumed to provide comparable results if results of cPDR_90min_ differ by less than 10% (Δ_cPDR_ = 0.6%). Estimating a standard deviation of 1.2, the effect size was determined as dz = 0.56. We estimated that n = 36 would be sufficient to provide a power (1- β) of 0.9 with α = 0.05. During a pre-specified interim analysis after 26 included subjects, a *post hoc* power analysis revealed an actual power (1- β) of 1.0 for the primary endpoint, substantially exceeding the pre-specified difference threshold. Given the large effect size (Coehn’s d = 3.2) and the high statistical significance achieved, continued enrollment to n = 36 was unlikely to substantially modify conclusions about primary kinetic differences and recruitment was stopped.

#### 2.9.2 Statistical considerations

The primary endpoint was analyzed using *t*-test for paired samples. cPDR_0min_ - cPDR_90min_ was analyzed using repeated measures analysis of variance (RM-ANOVA) with Šídák’s multiple comparisons test for *post hoc* analysis.

Categorical data was analyzed using Fisher´s exact test or χ^2^ test. Continuous normally distributed data was analyzed using *t*-test for paired or unpaired samples (the latter with Welch’s correction), analysis of variance or linear models in general. In addition, correlations are to be calculated using Pearson correlation. A linear regression model was used to determine the crossing of the slope of the combined test with the cPDR_90min_ of the ^13^C-methionine breath test. Results for crossing of curves are presented as mean [95% confidence interval]. Interval between study days is presented as median (min; max). Results are presented as means ± standard error of mean (SEM). Descriptive data is presented as mean ± standard deviation. Statistical significance was defined as p < 0.05. Statistical analyses were performed using GraphPad Prism Version 10 for Windows (GraphPad Software, San Diego, California USA, www.graphpad.com).

## 3 Results

From February until October 2023, we screened 32 subjects and included 26 participants (reasons for exclusion: withdrawal of consent after completing first study visit [five subjects], diagnosed T2D at screening [one subject]). Baseline parameters of completers are presented in **Table 1**. Subjects were mostly male (56.7%), mean age was 61 ± 15.9 years, and all subjects were overweight (17 [65.4%]) or obese (WHO-grade I adiposity: seven [26,9%], WHO-grade II adiposity: two [7.7%], mean BMI: 29.3 [± 3.6] kg/m²). Mean FIB-4 score was 1.4 ± 1.1 and two (7.7%) subjects had scores indicating a high risk of fibrosis (**Supplementary Figure S1**) (23).

**Table 1:** Baseline characteristics of study participants.

Parameter	Unit	Normal limits	Result
Clinical parameters and medical history			
Age	years	n/a	61 ± 15.9
Sex	female/male (%female)	n/a	11/15 (42.3%)
Body Mass Index	kg/m²	< 25.0	29.3 ± 3.6
Arterial hypertension	n (%)	n/a	14 (53.8%)
Atrial fibrillation	n (%)	n/a	1 (3.8%)
Previously diagnosed steatotic liver disease	n (%)	n/a	4 (15.4%)
Laboratory parameters			
HbA_1C_	%	4.8 - 6.0	5.6 ± 0.3
	mmol/L	29 - 42	38.0 ± 3.7
Creatinine clearance*	ml/min/1.73	> 90	79.4 ± 22.7
GGT	U/L	10 - 71	27.2 ± 18.2
AST	U/L	10 - 50	25.8 ± 6.4
ALT	U/L	10 - 50	25.3 ± 9.6
Bilirubin	mg/dL	< 1.2	0.5 ± 0.3
	μmol/L	< 20.52	8.55 ± 5.13
CRP	mg/dL	< 5	5.2 ± 0.6
Albumin	g/L	3.5 - 5.2	4.4 ± 0.3
INR	n/a	0.8 - 1.1	1.0 ± 0.1
FIB-4 score	n/a	< 2.67	1.4 ± 1.1
High-risk FIB-4	n (%)	> 2.67	2 (7.7%)
Medication			
Antihypertensives	n (%)	n/a	16 (61.5%)
Antiplatelet drugs	n (%)	n/a	6 (23.1%)
Anticoagulation	n (%)	n/a	2 (7.7%)
Statin	n (%)	n/a	9 (34.6%)

Data are presented as means ± standard deviation, or number and percentage (%) of total. *According to the formula defined by the Chronic Kidney Disease Epidemiology Collaboration (CKD-EPI). HbA_1C_, hemoglobin A1c; GGT, Gamma-glutamyl transferase; AST, Aspartate Aminotransferase; ALT, Alanine transaminase; ALP, Alkaline phosphatase; CRP, C-reactive protein; INR, international normalized ratio; FIB-4, Fibrosis-4 Index.

Further characteristics are displayed in **Supplementary Table S3** and **S4**.

Arterial hypertension was diagnosed in 14 (53.8%) subjects and four (15.4%) had previously confirmed steatotic liver disease. None of the subjects were diagnosed with diabetes mellitus, but ten (38.5%) had prediabetes according to HbA_1c_ (mean HbA_1c_ 5.6 ± 0.3%). History of cardiovascular disease was present in two subjects (stroke). The tests were conducted with a median interval of 9.5 (1–75) days.

### 3.1 ^13^C-methionine breath test

#### 3.1.1 Kinetics

**Figure 1:**
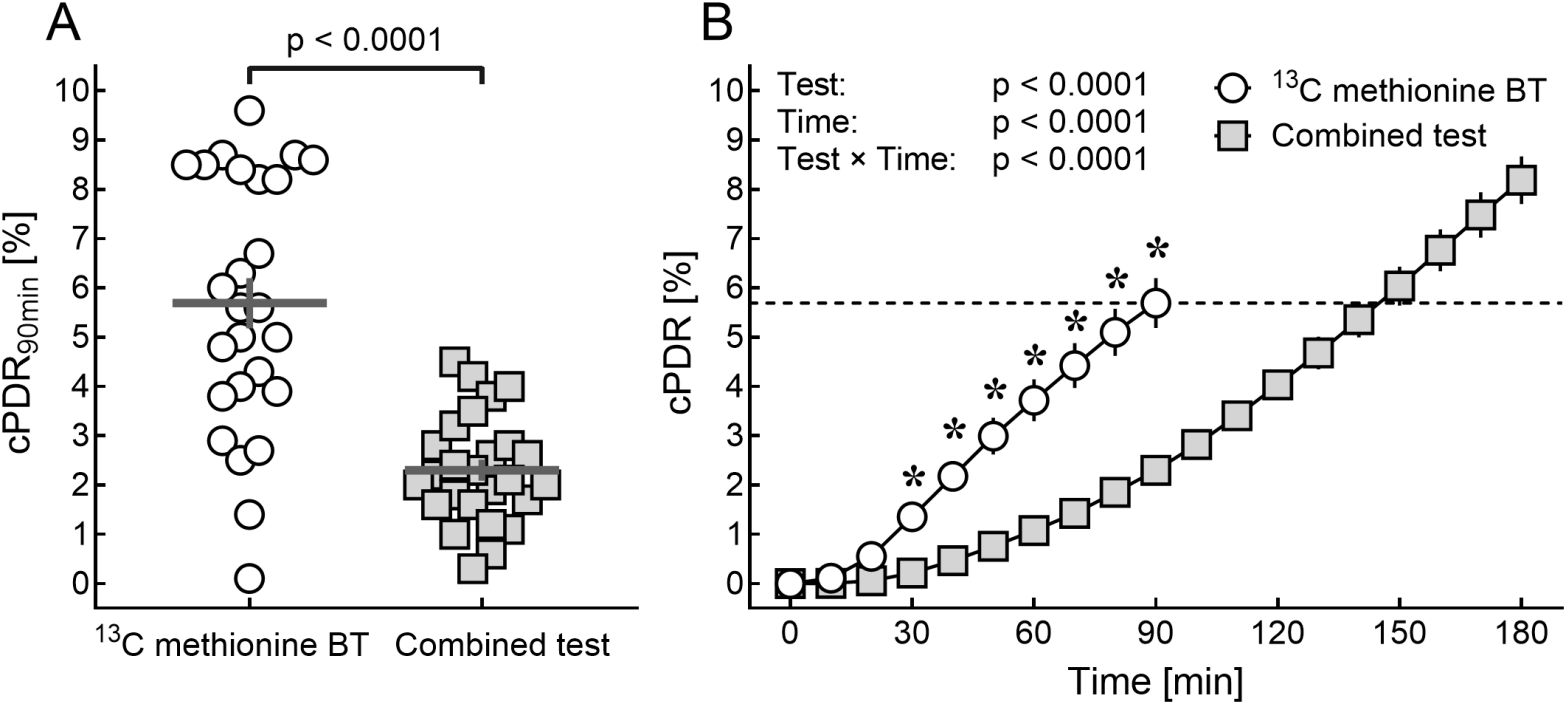
Cumulative percentage dose rate (cPDR) of the ^13^C-methionine breath test and the combined test. **(A)** Shows the cumulative percentage dose rate (cPDR) after 90 min of the ^13^C-methionine breath test (BT; white circles) and the combined test (grey squares). The horizontal grey lines in panel A display the mean, the vertical grey lines display the standard error of mean (SEM). Data was analyzed using Fisher’s *t*-test for paired samples. **(B)** Shows the cPDR over time. Data from 0–90 min (cPDR_0min_ – cPRD_90min_) was analyzed using repeated measures analysis of variance (RM-ANOVA) with Bonferroni’s correction for *post hoc* analysis. Significant differences in time points (p < 0.05) were marked with an asterisk. The dashed line marks the mean cPDR of the ^13^C-methionine BT at 90 minutes (cPDR_90min_ = 5.7%).

After 90 minutes, cPDR was significantly lower in the combined test as compared to the ^13^C-methionine breath test (**Figure 1A**, cPDR_90min_ 2.3 ± 0.2% vs. 5.7 ± 0.5%; p < 0.0001). The slope for cPDR was significantly lower in the combined test, with the slope of the combined test showing a delayed, almost linear increase after 100 min (**Figure 1B**). Linear regression analysis revealed that the slope of the combined test from 110–180 min (y = 0.069. x – 4.205, r² = 0.999, p < 0.0001) crossed the cPDR_90min_ of the methionine breath test at 144.3 [137.8 to 151.0] min. The estimated cPDR_145min_ ([cPDR_150min_ – cPDR_140min_]: 2 + cPDR_140min_) of the combined test (5.7 ± 0.4%) was practically identical to the cPDR_90min_ of the methionine test (Δ_cPDR_ 0.0038 ± 0.47%, p = 0.99).

#### 3.1.2 Clinical outcomes

According to the cPDR_90min_ in the ^13^C-methionine breath test, 18 (69.2%) subjects had results consistent with absence of MASH or fibrosis. In 3 (11.5%) subjects, MASH was present (cPDR_90min_ < 4.2%) and liver fibrosis of higher degree (F2-F3, cPDR_90min_ < 3.65%) was found in 5 (19.2%). Two subjects with an abnormal ^13^C-methionine breath test also had an abnormal FIB-4 score indicating high probability of fibrosis (**Supplementary Figure S1**).

As exploratory analysis, the established cPDR cut-offs for the ^13^C-methionine breath test after 90 minutes were applied to interpretate the cPDR_145min_ of the combined test. Based on the cPDR_145min_, 21 (80.8%) subjects had results consistent with absence of MASH or fibrosis and 5 (19.2%) had fibrosis. The outcome did not differ significantly between cPDR_90min_ and cPDR_145min_ (p = 0.20). In 6 (23.1%) subjects, the results varied between the two tests (healthy [single test] to fibrosis [combined test]: 2 [7.7%], healthy [single test] to MASH [combined test]: 3 [11.5%]; fibrosis [single test] to healthy [combined test]: 1 [3.8%]). The only parameter that differed significantly between subjects with a different outcome in combined as compared to the single ^13^C-methionine breath test and those with an unchanged outcome was plasma bilirubin concentration (0.8 ± 0.1 vs. 0.5 ± 0 mg/dL, p = 0.037). Further characteristics are displayed in **Supplementary Table S2**.

### 3.2 Oral glucose tolerance test

#### 3.2.1 Glucose

**Figure 2:**
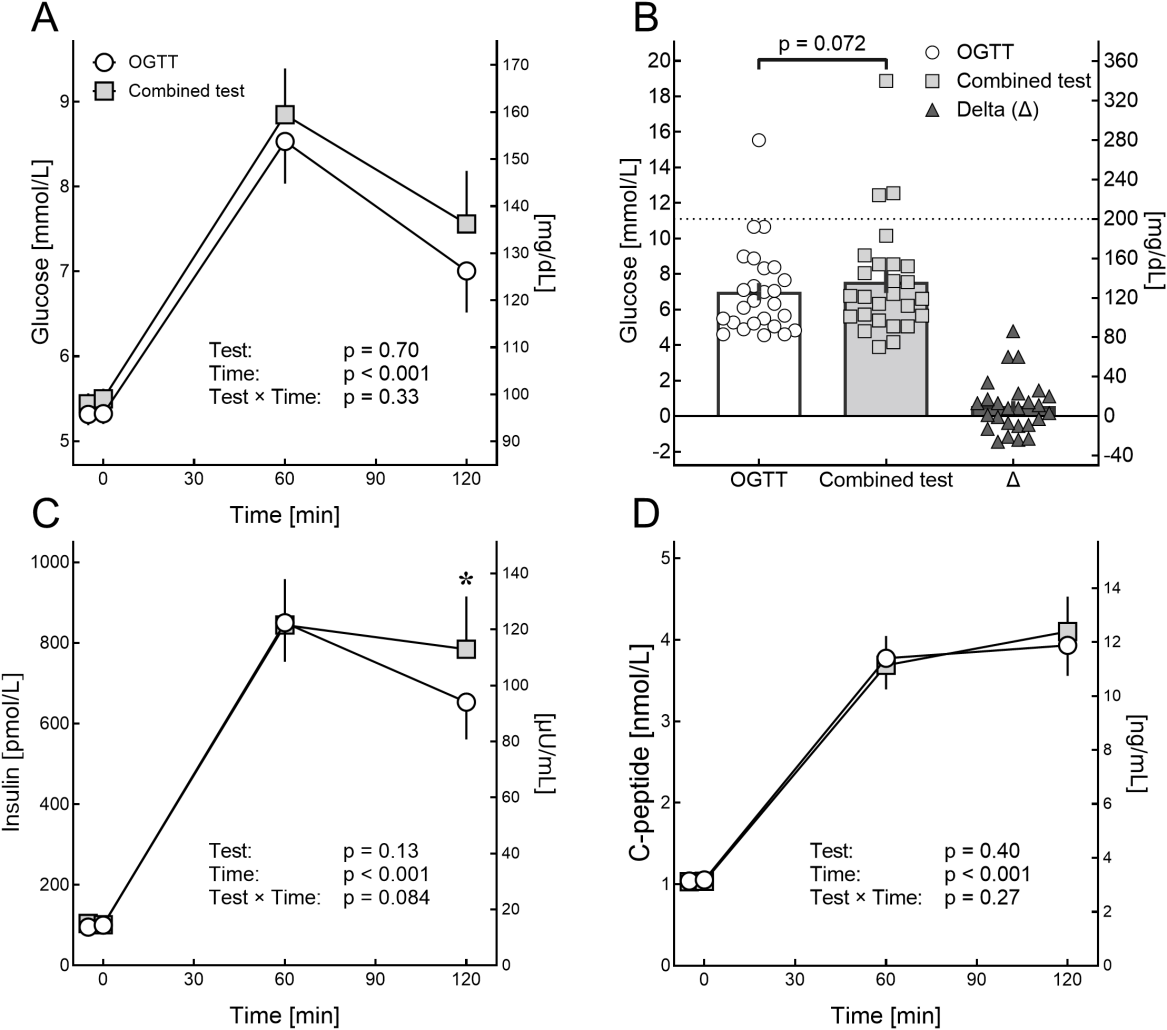
Results of the oral glucose tolerance test with and without the ^13^C-methionine breath test. Plasma concentrations of glucose **(A, B)**, insulin **(C)** and C-peptide **(D)** after a 75 g oral glucose challenge (oral glucose tolerance test) alone (white circles) or combined with a ^13^C-methionine breath test (grey squares). Error bars display standard error of mean (SEM). **(B)** Displays the plasma glucose concentration after 120 minutes for both tests and their delta (Δ) with the P-value being calculated using Student’s *t*-test for paired samples. Data in **(A, C, D)** was analyzed using baseline subtracted 2-way analysis of variance (ANOVA) with “Test” representing the overall effect of the intervention, “Time” representing the effect of time and “Test x Time” representing the interaction of intervention and time on plasma concentrations. If “Test” or “Time” were significant (p < 0.05), *post hoc* (Šídák’s) tests were performed to locate significant differences to single time points (asterisks).

Plasma glucose concentrations at baseline (Δ 0.15 ± 0.01 mmol/L [2.7 ± 1.4 mg/dL], **Figure 2A**) and after 120 minutes (Δ 0.56 ± 0.29 mmol/L [10.0 ± 5.3 mg/dL], **Figure 2B**) were by trend higher in the combined test. Plasma glucose concentrations after 60 minutes were not significantly different (Δ 0.31 ± 0.26 mmol/L [5.5 ± 4.7 mg/dL]).

#### 3.2.2 Insulin and C-peptide

Insulin at baseline (Δ 4.0 ± 4.9 pmol/L [0.58 ± 0.71 µU/mL]) and after 60 minutes (Δ 28.8 ± 60.6 pmol/L [4.14 ± 8.72 µU/mL]) did not differ between the tests (**Figure 2C**). However, after 120 min plasma insulin concentrations were significantly higher in the combined test (Δ 131.1 ± 57.9 pmol/L [18.87 ± 8.33 µU/mL]). For C-peptide, no significant difference was found (baseline: Δ 0.02 ± 0.04 nmol/L [0.06 ± 0.12 ng/mL, 60 minutes: Δ 0.03 ± 0.13 nmol/L [0.08 ± 0.4 ng/mL], 120 minutes: Δ 0.17 ± 0.13 nmol/L [0.52 ± 0.4 ng/mL]; **Figure 2D**). Results for HOMA IR (3.5 ± 0.5 vs. 3.5 ± 0.5) and HOMA β (127.8 ± 18.0 vs. 161.1 ± 15.7) did not differ between the tests (p = 0.78 and p = 0.11, respectively).

#### 3.2.3 Clinical outcomes

Overall, no statistically significant differences between the outcome of the single OGTT and the combined test were found (**Table 2**). However, the number of subjects diagnosed with diabetes was numerically higher in the combined test (1 [3.8%] vs. 3 [11.5%], p = 0.61). Details of the subjects diagnosed with diabetes are presented **Supplementary Table S5**.

**Table 2:** Interpretation of the plasma glucose concentrations at 120 min in the oral glucose tolerance test and the combined test.

Result of the test	OGTT	Combined test	p-value
No diabetes	16 (61.5%)	12 (46.2%)	0.40
Impaired fasting glucose	3 (11.5%)	8 (30.8%)	0.17
Impaired glucose tolerance	6 (23.1%)	6 (23.1%)	> 0.99
Both IGT and IFG	0	3 (11.5%)	0.24
Diabetes	1 (3.8%)	3 (11.5%)	0.61
Plasma glucose ≥ 8.6 mmol/L (155 mg/dL)	12 (46.2%)	10 (38.5%)	0.78

Data are presented as number and percentage (%) of total. OGTT, oral glucose tolerance test; IGT, impaired glucose tolerance; IFG, impaired fasting glucose. Statistics, Student’s *t*-test for paired samples.

#### 3.2.4 Safety outcomes

No adverse or serious adverse events occurred. All 26 subjects completed all three study visits. No episodes of dizziness, nausea, sleepiness, polyuria, or significant blood pressure changes were reported. Some patients reported an unusual taste after ingestion of the ^13^C-methionine, not fulfilling the criteria of an adverse event.

## 4 Discussion

The present study aimed to determine if the combination of a 75 g oral glucose tolerance test and a ^13^C-methionine breath test alters methionine kinetics and provides results comparable to single-test protocols. The primary finding is that the result of the breath test (cPDR_90min_) significantly differs between the single and the combined. However, the cPDR_145min_ of the combined test provides comparable results to the cPDR_90min_ of the single test to assess the mitochondrial function of hepatocytes. Furthermore, the diagnostic outcome of the oral glucose challenge is mostly consistent between the tests.

In the combined test, the cPDR_90min_ was significantly lower than the cPDR_90min_ of the single ^13^C-methionine breath test (**Figure 1A**). The curve is clearly less steep, suggesting a delayed metabolism or absorption of ^13^C-methionine. A potential mechanism for the delayed increase in cPDR could be the metabolic challenge for hepatocytes associated with the glucose challenge. In contrast to this theory, no influence of methionine on glucose-6-phosphatase *in vitro* (primary bovine neonatal hepatocytes) was found, however, mitochondrial phosphoenolpyruvate carboxykinase expression was marginally decreased (24). The most likely reason for the delayed increase in exhaled ^13^C appears to be delayed gastric emptying, induced by the caloric load of the OGTT. The velocity of gastric emptying is a major determinant of the postprandial rise in plasma glucose (25). Importantly, it is dependent on the size and composition of meal with higher caloric content significantly slowing the rate of gastric emptying (26). The increase of macronutrient content of the solution by adding glucose certainly decelerated gastric emptying in the present study, resulting in a delayed absorption of the ^13^C-methionine in the small intestine, thus a delayed hepatic metabolism and ultimately a delayed ^13^C-exhalation. While there is a large interindividual variation in the velocity of gastric emptying (especially in obesity and diabetes (27)), the intraindividual variation is less pronounced. The velocity of gastric emptying decreases with increasing caloric content of the ingested food, ranging from 1–4 kcal per minute (28), with differences between women and men and different ethnic groups. However, since gastric emptying was not assessed in the present study, this mechanism, although being highly likely, remains speculative. Future studies should evaluate alternative or complementary pathways explaining the observed effect, including altered intestinal absorption, acute hepatic metabolic competition, hormonal modulation by GLP-1, GIP and insulin or microbiota-related changes.

The ^13^C-methionine breath test quantifies hepatic mitochondrial dysfunction but may not capture all effects of MASLD on cardiovascular disease and risk (3, 5). Beyond hepatic mitochondrial assessment, patients with preserved ^13^C-methionine breath test results may still be at significant cardiovascular risk (quantified for example by an carotid wall thickening) due to subclinical inflammation (29). Conversely, abnormal breath test results strongly predict increased carotid artery wall thickness and cardiovascular risk. Consequently, the combined OGTT/^13^C-methionine test should be understood as potential screening tool and certainly does not replace comprehensive cardiovascular risk assessment (including parameters like carotid media thickness, lipid profile, blood pressure) in MASLD patients (30).

The combined test protocol showed an excellent safety profile at the 2 mg/kg methionine dose. Unlike prior studies using higher doses (100 mg/kg), which reported substantial dizziness, nausea, somnolence, polyuria, and hemodynamic changes (31), none of these adverse effects occurred here, confirming favorable tolerability. Combined with the non-invasive nature of both tests, this supports further studies investigating real-world clinical feasibility for metabolic screening.

Overall, the oral glucose tolerance tests were not significantly different between the tests (**Figure 2**). However, the trend to higher plasma glucose concentrations after 120 min with an increase of ~ 4.1 ± 3.5% (mean ± SEM) versus the single test must be considered. Both, postprandial plasma glucose concentrations and the rate of gastric emptying are depending on the level of glycaemia (27). Since plasma glucose concentrations at baseline were higher in the combined test (~ 2.6 ± 1.3% [mean ± SEM]), the small difference in baseline plasma glucose concentration might have impacted the plasma glucose concentrations during the test. In contrast, an influence of the caloric content of methionine (approximately 1.7 kJoule [0.4 kcal]) per 100 mg, corresponding to ~ 0.1 g glucose) is rather unlikely. Other factors known to influence plasma glucose excursions after an oral glucose challenge (i.e., diet, activity level, stress, sleep, or physiological variability including changes in body weight) could potentially influence the outcome of glucose tolerance tests. These factors were not assessed systematically. While these influences explain the slight intertest variability, the overall result remained unchanged. Also, C-peptide, HOMA IR and HOMA β were consistent between the tests. This confirms the validity of the combined test as compared to an OGTT as one standard for the diagnosis of T2D. When discussing the results of the present study and deviations between the single tests and the combined test, the sensitivity and specificity of each test must be considered. Since neither the OGTT nor the ^13^C-methionine breath test provide a 100% sensitivity and specificity, the differences observed in the present study may easily be explained by the limitations of the individual test.

Liver biopsy remains the only method to allow for definite diagnosis of metabolic liver disease including steatohepatitis and the only method to evaluate relevant microscopic features like ballooning or lobular inflammation (23). However, current guidelines also appreciate that liver biopsy is not required for clinical management of individuals with MASLD in most cases (23). Currently, several non-invasive options to assess liver function, steatosis, fibrosis and cirrhosis in subjects with metabolic syndrome/suspected MASLD are available (32). These include serum biomarkers (33) and elastography utilizing ultrasound or magnetic resonance imaging (MRI) techniques (34). However, some tests may be difficult to perform in severely obese subjects, cases with contraindication for MRI or in subjects with rib-injuries (34). The advantages of ^13^C breath tests are that they examine *in vivo* the hepatic mitochondrial function and therefore offer a different approach to assessment of liver diseases compared to the above-mentioned methods. Furthermore, breath tests are cost-effective (e.g., compared to MRI) and can easily be implemented in clinical practice (35). The test is non-invasive and non-radioactive. Impaired liver function from various causes, such as drug-related acute liver toxicity, oxidative stress and impaired hepatic mitochondrial oxidation, can be detected (36). Since mitochondria are essential for fatty acid oxidation, lipogenesis and gluconeogenesis, their dysfunctionality is appreciated as key factor in the pathophysiology of several metabolic diseases (8). It should also be appreciated that obesity and metabolic syndrome are risk factors for both, T2D and MASLD (37). Consequently, the advantage of combining a screening method for both diseases is obvious. However, while the present approach allows for easy follow-up or routine liver function testing it was not formally validated in the present pilot study and needs further evaluation in an independent cohort or alongside established MASLD and diabetes diagnostic comparators.

The present study has limitations that should be considered. The study design was not blinded to the participant or the investigators. Also, clinical and laboratory differences between the tests were not assessed. Given the maximum interval between tests of up to 75 days, these differences could have a potential impact on the tests results (e.g., the weight loss of one participant most probably leading to an improved OGTT result). However, most tests were conducted within 11 days and significant clinical or laboratory changes in this interval appear unlikely. Of note, the present study did not evaluate the efficacy and reliability of the ^13^C-methionine breath test for diagnosing MAFLD or assessing liver function in comparison to other non-invasive diagnostic procedures or liver biopsy. While this was not the aim of the present study, no further conclusions on the performance of the ^13^C-methionine breath test to other non-invasive methods can be drawn. Furthermore, adding an intervention to investigate changes in cPDR_90min_ would have provided valuable insights to both, the ^13^C-methionine breath test and the combined test. This pilot study evaluated middle-aged to older subjects (mean age 61 ± 16 years), reflecting peak disease prevalence. While appropriate for protocol validation, future studies should target younger individuals with early metabolic dysfunction for early intervention and prevention strategies. Despite these limitations, we believe the results of this study warrant further investigation in a larger patient population. This could include a randomized controlled trial with measurements taken both before and after an intervention, as well as comparisons with other invasive and non-invasive methods for assessing hepatic function and metabolic liver disease.

In summary, this pilot study demonstrates that combining a ^13^C-methionine breath test with a 75g OGTT substantially alters ^13^C-methionine kinetics, potentially due to delayed gastric emptying, while the OGTT diagnostic categorization remains preserved. When breath collection is extended to 145 minutes, cPDR values approach comparability to single-test protocols. These findings support the feasibility of a time-adjusted combined protocol for simultaneous metabolic assessment. Further studies are necessary to independently assess the validity of cPDR_145min_ of the combined test compared to other liver assessment tools (e.g., transient elastography or liver biopsy) and formal validation against imaging/elastography standards, diagnostic accuracy quantification, and prospective outcome studies are required before clinical implementation as a screening tool.

## Data Availability

The data that support the findings of this study are available from the corresponding author, DRQ, upon reasonable request.
