# Supplementary material for: A pilot study on a combined non-invasive screening test for metabolic dysfunction-associated steatotic liver disease and type 2 diabetes
Source: Front Endocrinol (Lausanne). 2026 Feb 12;17:1780005. doi: 10.3389/fendo.2026.1780005 (PMC12935636; doi:10.3389/fendo.2026.1780005)
Supplement: Supplementary file 1 [file DataSheet1.pdf]

A Pilot Study on a Combined Non-invasive Screening Test for Metabolic Dysfunction-Associated Steatotic Liver Disease and Type 2 Diabetes

Supplementary Material

Supplementary Table S1. Full inclusion and exclusion criteria.
Inclusion criteria:
• Informed consent
• Body mass index (BMI) ≥ 25 kg/m²
Exclusion criteria:
• Lack of consent
• Pregnancy
• Conditions that could affect the altered absorption of ^{13}C-methionine or glucose from the gastrointestinal tract (e.g., previous gastrointestinal tract surgery [except cholecystectomy and appendectomy], chronic inflammatory bowel diseases)
• Diabetes mellitus (any subtype)
• Autoimmune hepatitis
• Acute or chronically active viral hepatitis (e.g., hepatitis B, hepatitis C)
• Toxic hepatitis (especially alcohol-induced and/or drug-induced hepatitis)
• Alcohol consumption of more than 10g/day for women or more than 20g/day for men
• Chronic pulmonary disease with suspected impaired exhalation of CO_2

Supplementary Table S2. Clinical characteristics of subjects with a divergent outcome in the combined test vs. those with the same result					
Parameter	Unit	Normal limits	Divergent outcome	Unchanged outcome	p-value
Clinical parameters and medical history					
Age	years	n/a	62 ± 5	61 ± 3.8	0.83
Sex	female (%female)	n/a	3 (50 %)	8 (40 %)	>0.99
Body Mass Index	kg/m²	< 25.0	28.2 ± 1.3	29.6 ± 0.8	0.38
Arterial hypertension	n (%)	n/a	3 (50 %)	11 (55 %)	>0.99
Atrial fibrillation	n (%)	n/a	1 (16.7 %)	0	0.23
History of pancreatitis	n (%)	n/a	0	5 (25 %)	0.30
History of Steatosis hepatis	n (%)	n/a	0	4 (20 %)	0.54
Laboratory parameters					
HbA_{1C}	%	4.8 - 6.0	5.6 ± 0.1	5.6 ± 0.1	0.84
	mmol/L	29 - 42	37.7 ± 1.4	38.0 ± 0.9	0.84
Creatinine clearance*	ml/min/1.73	> 90	75.0 ± 11.9	80.7 ± 4.7	0.67
GGT	U/L	10 - 71	42.0 ± 11.9	22.8 ± 2.5	0.17
	µkat/L	0.17-1.18	0.7 ± 0.19	0.4 ± 0.04	
AST	U/L	10 - 50	29.8 ± 4.3	24.6 ± 0.9	0.27
	µkat/L	0.17-0.83	0.5 ± 0.07	0.4 ± 0.02	
ALT	U/L	10 - 50	26.8 ± 3.9	24.8 ± 2.2	0.66
	µkat/L	0.17-0.83	0.4 ± 0.07	0.4 ± 0.04	
Bilirubin	mg/dL	< 1.2	0.8 ± 0.1	0.5 ± 0	0.037
	µmol/L	<20.52	13.7 ± 1.71	8.55 ± 0	
CRP	mg/dL	< 5	5.3 ± 0.3	5.1 ± 0.1	0.61
	mg/L	<50	530 ± 3	51 ± 1	
Albumin	g/L	3.5 - 5.2	4.5 ± 0.2	4.4 ± 0.1	0.74
INR		0.8 - 1.1	1.1 ± 0.0	1.0 ± 0	0.18
Medication					
Antihypertensives	n (%)	n/a	2 (33.3 %)	14 (70 %)	0.16
Antiplatelet drugs	n (%)	n/a	2 (33.3 %)	4 (20 %)	0.60
Anticoagulation	n (%)	n/a	0	2 (10 %)	>0.99
Statin	n (%)	n/a	1 (16.7 %)	8 (40 %)	0.38
Results of the 75 oral glucose challenge					
Diabetes	n (%)	n/a	1	0	0.23
HOMA IR			2.9 ± 0.9	3.8 ± 2.8	0.15
HOMA B			129.4 ± 57.1	170.9 ± 85.4	0.15
Results of the 75 oral glucose challenge when combined with the ^{13}C methionine breath test					
Diabetes	n (%)	n/a	1	2	>0.99
HOMA IR			4.1 ± 1.4	3.3 ± 3.0	0.55
HOMA B			125.2 ± 31.7	123.5 ± 101.7	0.55

Data are presented as means ± standard deviation, or number and percentage (%) of total. *According to the formula defined by the Chronic Kidney Disease Epidemiology Collaboration (CKD-EPI). HbA_{1C} = hemoglobin A1c, GGT = Gamma-glutamyl transferase, AST = Aspartate Aminotransferase, ALT = Alanine transaminase, CRP = C-reactive protein, INR = international normalized ratio, HOMA-IR = Homeostatic Model Assessment fpr Insulin Resistance, HOMA B = Homeostasis Model Assessment of Beta-cell function

Supplementary Table S3. Baseline characteristics of study participants including dropouts.		
Parameter	Unit	All participants
Age	years	61.6 ± 16.2
Gender	female/male (%female)	11/20 (35.5 %)
BMI	kg/m^2	29.3 ± 3.5
Medication		
Antihypertensives	n (%)	18 (60 %)
Platet aggregation inhibitors	n (%)	7 (23.3 %)
NOAC	n (%)	1 (3.3 %)
Marcumar	n (%)	2 (6.7 %)
Statins	n (%)	9 (30 %)
Other lipid reducers	n (%)	2 (6.7 %)
Analgetics	n (%)	5 (16.7 %)
Antibiotics	n (%)	0
Antidepressants	n (%)	1 (3.3 %)
Anticonvulsants	n (%)	1 (3.3 %)
Medical history		
Steatosis hepatis	n (%)	4 (15.4 %)
Arterial hypertension	n (%)	16 (53.3 %)
Hypercholesterolemia	n (%)	5 (16.7 %)
Peripherial artery disease	n (%)	0
History of myocardial infarction	n (%)	0
History of stroke	n (%)	3 (10 %)
Cardiac arrhythmias	n (%)	2 (6.7 %)
Atrial fibrillation	n (%)	1 (3.3 %)
Chronic heart failure	n (%)	0
Arteriosclerosis	n (%)	1 (3.3 %)
Valvular heart disease	n (%)	2(6.7 %)
History of pancreatitis	n (%)	5 (16.7 %)
Smoking	n (%)	2 (6.7 %)
Data are presented as mean ± SD, or number and percentage (%) of patients, SD = standard deviation, BMI= Body Mass Index, NOAC = non-vitamin K antagonist anticoagulant PAD = peripheral artery disease		

Supplementary Table S4. Baseline laboratory results of study participants including dropouts.

Parameter	Unit	Normal limits	All participants
Leucocytes	/µL	4600 - 9500	6636.5 ± 1937.9
	Gpt/L	4.6 - 9.5	6.6 ± 1.9
Hemoglobin	g/dL	14 - 18	14.2 ± 1.3
	mmol/L	8.69 - 11.17	8.81 ± 0.81
Thrombocytes	/µL	150000 - 400000	240500 ± 48323.3
	Gpt/L	150 - 400	240.5 ± 48.3
HbA_{1C}	%	4.8 - 6.0	5.6 ± 0.3
	mmol/L	29 - 42	38.0 ± 3.7
Urea	mg/dL	17 - 49	41.1 ± 34.3
	µmol/L	2.84 - 8.18	6.86 ± 5.73
Creatinine	mg/dL	0.7 - 1.20	1.0 ± 0.3
	µmol/L	61.88 - 106.08	88.4 ± 26.52
Creatinine clearance	ml/min/1.73	> 90	79.4 ± 22.7
LDL	mg/dL	69 - 149	111.7 ± 37.3
	mmol/L	1.53 - 3.87	2.9 ± 0.97
HDL	mg/dL	33 - 84	59.2 ± 20.8
	mmol/L	0.86 - 2.18	1.54 ± 0.54
Lp (a)	mg/dL	< 75	55.2 ± 58.1
ntBNP	pg/ml	< 125	161.1 ± 221.4
	pmol/L	< 14.75	19.01 ± 26.125
Lipase	U/L	13 - 60	45.0 ± 40.6
	µkat/L	0.22 - 1	0.75 ± 0.68
GGT	U/L	10 - 71	27.2 ± 18.2
	µkat/L	0.17 - 1.18	0.45 ± 0.3
AST	U/L	10 - 50	25.8 ± 6.4
	µkat/L	0.17 - 0.83	0.43 ± 0.11
ALT	U/L	10 - 50	25.3 ± 9.6
	µkat/L	0.17 - 0.83	0.42 ± 0.16
Cholinesterase	U/L	5320 - 12920	8070.1 ± 2317.3
	µkat/L	88.68 - 215.4	134.5 ± 38.6
ALP	U/L	35 - 104	77.5 ± 16.3
	µkat/L	0.58 - 1.73	1.29 ± 0.27
Bilirubin	mg/dL	< 1.2	0.5 ± 0.3
	µmol/L	< 20.52	8.55 ± 5.13
Sodium	mmol/L	136 - 145	137.0 ± 13.4
Potassium	mmol/L	3.50 - 5.10	9.7 ± 26.2
CRP	mg/dL	< 5.0	5.2 ± 0.6
	mg/L	< 50	52 ± 6
Albumin	g/L	3.5 - 5.2	4.4 ± 0.3
Serum protein	g/L	66 - 87	70.2 ± 3.8
INR		0.8 - 1.1	1.0 ± 0.1
Quick	%	>75	93.6 ± 11.0
PTT	sec	26 - 40	29.5 ± 4.2

Data are presented as mean ± standard deviation, HbA_{1C} = hemoglobin A1c, LDL= low-density lipoprotein, HDL = high-density lipoprotein, Lp (a) = lipoprotein a, ntBNP = B-type natriuretic peptide, GGT = Gamma-glutamyl transferase, AST = Aspartate Aminotransferase, ALT = Alanine transaminase, ALP = Alkaline phosphatase, CRP = C-reactive protein, INR = international normalized ratio, PTT = partial thromboplastin time

Supplementary Table S5. Characteristics of subjects diagnosed with diabetes in the OGTT or the combined test

**Parameter**		**Unit**	**Subject 1**	**Subject 2**	**Subject 3**
Age		years	66	67	72
Sex		n/a	Female	Female	Male
BMI		kg/m²	26.8	30.1	34.7
HbA_{1c}		mmol/L (%)	39.9 (5.8)	36.6 (5.5)	46.5 (6.4)
Plasma glucose concentrations					
Combined test	(baseline)	mmmol/L (mg/dL)	6.5 (117)	5.1 (91)	6.7 (120)
Combined test	(60 min)	mmmol/L (mg/dL)	14.2 (255)	10.9 (196)	12.2 (219)
Combined test	(120 min)	mmmol/L (mg/dL)	18.9 (340)	12.4 (224)	12.5 (226)
OGTT	(baseline)	mmmol/L (mg/dL)	5.3 (96)	5.1 (91)	7.2 (130)
OGTT	(60 min)	mmmol/L (mg/dL)	14.0 (253)	9.0 (163)	9.5 (172)
OGTT	(120 min)	mmmol/L (mg/dL)	15.5 (280)	7.7 (138)	10.7 (192)

Characteristics of the subjects diagnosed with diabetes based on the plasma glucose concentration after 120 min in at least one of the tests. Subject 1 was diagnosed with diabetes in both tests. Subject 2 had a significantly higher plasma glucose concentration at 120 min in the combined test. Further evaluation revealed that the subject had lost a significant amount of body weight (16 kg) between the tests by following an almost carbohydrate-free diet (BMI at follow-up: 25.5 kg/m²). Subject 3 had impaired glucose tolerance at the impaired test and almost reached the cut-off value for diabetes diagnosis. Of interest, all subjects would be diagnosed with diabetes based on the 1-hour plasma glucose concentration (> 8.6 mmol/L or > 155 mg/dL; Jagannathan et al. The Oral Glucose Tolerance Test: 100 Years Later. Diabetes Metab Syndr Obes. 2020 Oct 19;13:3787-3805).

Supplementary Figure S1: Results of the FIB-4 score

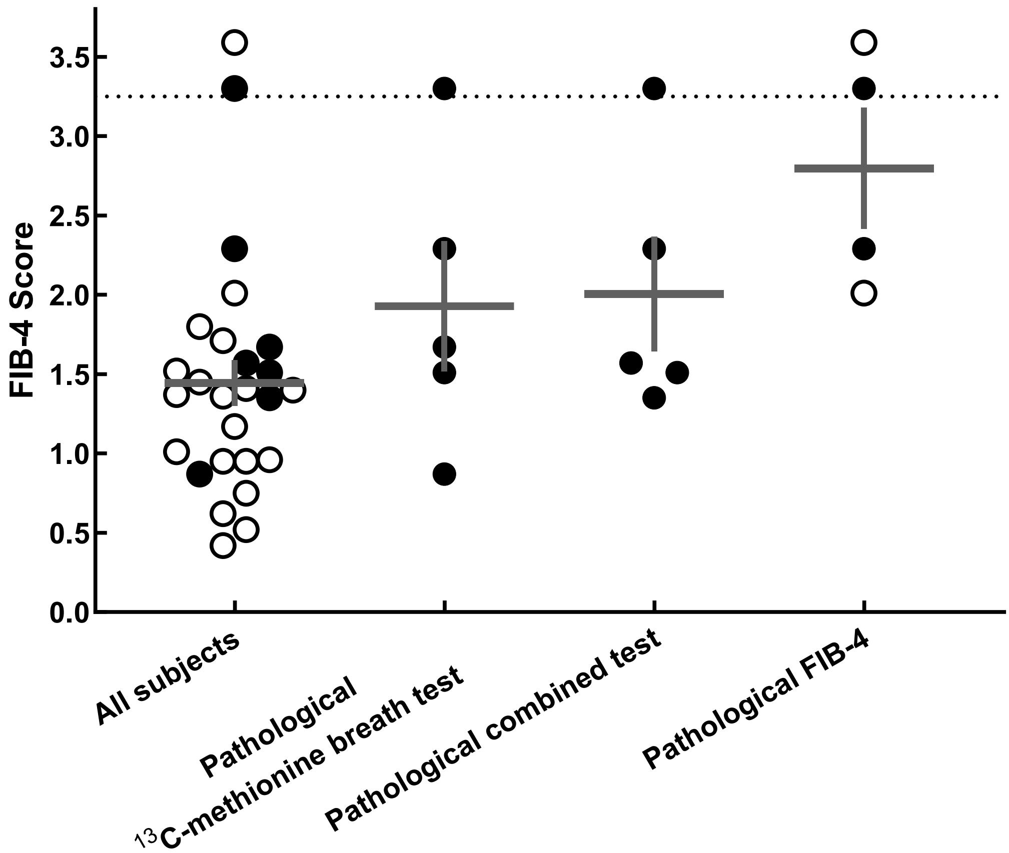

Results of the FIB-4 score for all subjects, and those with an abnormal ^{13}C-methionine breath test, an abnormal combined ^{13}C-methionine and oral glucose tolerance test, and those with only an abnormal (age-adjusted) result in the FIB-4 score. Subjects with at least one ^{13}C-breath test indicating fibrosis are represented by black circles. Subjects with a normal ^{13}C-methionine breath test, indicating no fibrosis, are represented by white circles. The dotted line indicates the cut-off for an abnormal FIB-4 score indicating a high risk of fibrosis. The grey lines indicate the mean ± standard error of the mean.
